# Estimating dynamic changes of tissue attenuation coefficient during high-intensity focused ultrasound treatment

**DOI:** 10.1186/2050-5736-1-14

**Published:** 2013-09-02

**Authors:** Siavash Rahimian, Jahan Tavakkoli

**Affiliations:** 1 Department of Physics, Ryerson University, 350 Victoria Street, Toronto, Ontario M5B 2K3, Canada

**Keywords:** HIFU, Thermal lesion, Treatment monitoring, Tissue attenuation coefficients, Contrast-to-speckle ratio

## Abstract

**Background:**

This study investigated the dynamic changes of tissue attenuation coefficients before, during, and after high-intensity focused ultrasound (HIFU) treatment at different total acoustic powers (TAP) in *ex vivo* porcine muscle tissue. It further assessed the reliability of employing changes in tissue attenuation coefficient parameters as potential indicators of tissue thermal damage.

**Methods:**

Two-dimensional pulse-echo radio frequency (RF) data were acquired before, during, and after HIFU exposure to estimate changes in least squares attenuation coefficient slope (Δ*β*) and attenuation coefficient intercept (Δ*α*_0_). Using the acquired RF data, Δ*β* and Δ*α*_0_ images, along with conventional B-mode ultrasound images, were constructed. The dynamic changes of Δ*β* and Δ*α*_0_, averaged in the region of interest, were correlated with B-mode images obtained during the HIFU treatment process.

**Results:**

At a HIFU exposure duration of 40 s and various HIFU intensities (737–1,068 W/cm^2^), Δ*β* and Δ*α*_0_ increased rapidly to values in the ranges 1.5–2.5 dB/(MHz.cm) and 4–5 dB/cm, respectively. This rapid increase was accompanied with the appearance of bubble clouds in the B-mode images. Bubble activities appeared as strong hyperechoic regions in the B-mode images and caused fluctuations in the estimated Δ*β* and Δ*α*_0_ values. After the treatment, Δ*β* and Δ*α*_0_ values gradually decreased, accompanied by fade-out of hyperechoic spots in the B-mode images. At 10 min after the treatment, they reached values in ranges 0.75–1 dB/(MHz.cm) and 1–1.5 dB/cm, respectively, and remained stable within those ranges. At a long HIFU exposure duration of around 10 min and low HIFU intensity (117 W/cm^2^), Δ*β* and Δ*α*_0_ gradually increased to values of 2.2 dB/(MHz.cm) and 2.2 dB/cm, respectively. This increase was not accompanied with the appearance of bubble clouds in the B-mode images. After HIFU treatment, Δ*β* and Δ*α*_0_ gradually decreased to values of 1.8 dB/(MHz.cm) and 1.5 dB/cm, respectively, and remained stable at those values.

**Conclusions:**

Δ*β* and Δ*α*_0_ estimations were both potentially reliable indicators of tissue thermal damage. In addition, Δ*β* and Δ*α*_0_ images both had significantly higher contrast-to-speckle ratios compared to the conventional B-mode images and outperformed the B-mode images in detecting HIFU thermal lesions at all investigated TAPs and exposure durations.

## Background

High-intensity focused ultrasound (HIFU) is a non- or minimally invasive modality for conducting high-temperature thermal therapy. HIFU has the capability to induce biological effects deep into the body by delivering acoustic energy at a distance from the source, and to generate a millimeter-size focal region [1]. The HIFU beam induces biological effects in the focal region via thermal and mechanical mechanisms.

This study is focused on tissue damage caused predominantly by thermal mechanisms of HIFU. In the thermal mechanism, the acoustic wave energy is converted to heat through a variety of effects such as viscous shearing effects and relaxation processes [2]. The typical HIFU beam can generate intensities in the range of a few hundred to a few thousand watts per square centimeter at the focal point [1]. The combination of tight focusing and high intensities generated by the HIFU beam results in the generation of high temperatures (in excess of 60°C in the tissue) at the focal spot, resulting in instantaneous cell death primarily through coagulative necrosis, without heating the surrounding tissues [3,4].

Monitoring HIFU therapies ensures that the target volume is completely treated (thermally damaged). In addition, it ensures the safety of sensitive structures near or outside the target volume. The monitoring and assessment methods employed for HIFU treatments are divided into two major categories. In the first category, the thermal damage and structural changes in tissue are detected and monitored. In the second category, also known as thermometry, the tissue temperature at the target volume is monitored, providing feedback to the interventionist or an automated system.

X-ray imaging, magnetic resonance imaging (MRI), and ultrasound imaging have all been used as noninvasive methods for monitoring and assessment of tissue thermal damage in HIFU surgeries. X-ray was the earliest imaging modality used for guidance and monitoring of HIFU [5]. However, it has the usual problem of exposing both the patient and the interventionist to high doses of ionizing radiation during rather long HIFU treatment procedures. Significant advances have so far been made in the application of MRI for guidance in thermal therapies and more specifically HIFU therapy [6]. However, low image acquisition speeds (low temporal resolution), high costs, requirement for the use of MRI-compatible parts, and fitting everything within the confined space of the MRI chamber have restricted the use of MRI for HIFU treatment monitoring and control.

Ultrasound imaging is an attractive and promising modality for real-time monitoring of HIFU treatments. The past and current states of ultrasound-guided HIFU were recently reviewed by Tavakkoli and Sanghvi [7].

One of the most straightforward methods of HIFU lesion detection and visualization is ultrasound B-mode imaging [8,9]. In the first studies in which ultrasound was used to monitor high-temperature thermal therapies, it was observed that due to the formation and presence of strongly scattering gas bubbles during HIFU treatment, a region with very high echogenicity usually appeared at or in the vicinity of the focal region. The echogenicity of the hyperechoic regions was quantified for the purpose of evaluating tissue thermal damage after each HIFU exposure [10]. However, it has been shown that the hyperechoic region is consistently smaller than the actual coagulated region in the tissue, and it has a different shape. Therefore, it is not an accurate indicator of tissue thermal damage. The use of B-mode imaging to monitor HIFU relies on the presence of cavitation and microbubble activities [11]. Cavitation and microbubble activities strongly correlate with unpredictable HIFU lesions [12], and in applications where avoidance of microbubble activities is desirable, relying on B-mode imaging will be problematic.

Tissue elastic modulus (stiffness) is another physical parameter that can be exploited for HIFU lesion detection. HIFU lesions can be significantly stiffer than normal tissue [13]. Therefore, changes in tissue elastic modulus can be used to estimate the size and location of HIFU lesions. A number of elastographic measurement techniques have been proven feasible for the detection and characterization of HIFU-induced lesions [14-16]. However, with the exception of acoustic radiation force imaging technique [16], they all require extra instruments to apply an external stress in order to create the desired strain in the tissue. In addition, a uniform application of the stress to deep-seated tissues and regions that are generally not accessible, along with contamination of elasticity maps by motion artifact distortions [17], have imposed further limitations on elastographic techniques.

Tissue temperature can be noninvasively estimated by detecting and measuring changes in speed of sound as a function of temperature [18-21]. However, factors such as nonlinearity [22,23], thermal expansion, the effect of different intensity levels, accuracy of *in vivo* measurements [24], lack of sufficient data tabulating the relationship between temperature and speed of sound for different types of tissue, and finally inter-patient variability [25] have so far imposed serious limitations on noninvasive temperature measurement using ultrasound.

Studies conducted on changes in ultrasound tissue attenuation coefficient as a function of temperature or thermal dose [24,26-28] have shown that there is a significant increase in tissue attenuation coefficient as the temperature of tissue rises and ultimately as the tissue coagulates. Frequency-dependent ultrasound attenuation and its changes as a function of temperature or thermal dose are potentially valuable methods for differentiating normal and thermally coagulated tissue. Therefore, measurement of change in ultrasound attenuation can be exploited to monitor HIFU therapies. Ophir et al. extensively discussed various methods of attenuation estimation using radio frequency (RF) backscattered ultrasound signals [29]. Using attenuation mapping, it was shown that a thermally coagulated region (HIFU-induced lesion) of an *ex vivo* liver tissue can be effectively detected [30]. Bevan and Sherar developed one algorithm based on detecting changes in the slope of the logarithm of the envelope of the RF backscattered ultrasound signals [31] and another one based on the shift in the center frequency of such signals [32] to generate attenuation maps for monitoring of high-temperature thermal therapies. Seip et al. conducted a comparison of a few detection algorithms, including attenuation change, for real-time monitoring and control of HIFU lesions in *in vivo* canine prostates using RF backscattered ultrasound signals [33]. Most recently Zhang et al. studied the feasibility of using attenuation estimation using RF backscattered ultrasound signals, along with other RF-based methods for the monitoring of HIFU in transparent tissue-mimicking phantoms and in *ex vivo* bovine liver [34].

Our study was designed to investigate the possibility that changes in the tissue attenuation of HIFU lesions can be estimated with respect to initial attenuation of normal tissue, using the acquired backscattered ultrasound RF data. The aim was to (1) design and develop a system (both hardware and software) capable of acquiring backscattered ultrasound RF data before, during, and after any given HIFU exposure; and (2) design and implement suitable signal processing algorithms capable of quantitatively characterizing changes in attenuation of tissue in locations where HIFU lesions are created, in real-time, using the acquired backscattered ultrasound RF data. Two frequency domain algorithms were developed, and the transient characteristics of tissue attenuation coefficient parameters before, during, and after HIFU exposure at different total acoustic power (TAP) values in *ex vivo* porcine muscle tissues were investigated. In addition, tissue attenuation maps were generated and were further correlated with B-mode images. Finally, the imaging performances of the attenuation maps in detecting HIFU lesions were compared with each other and with conventional B-mode images.

## Materials and methods

### Experimental setup

**Figure 1:**
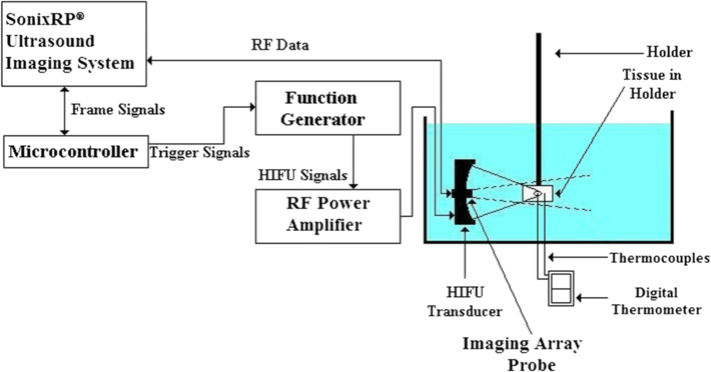
The image-guided HIFU experimental setup.

**Figure 2:**
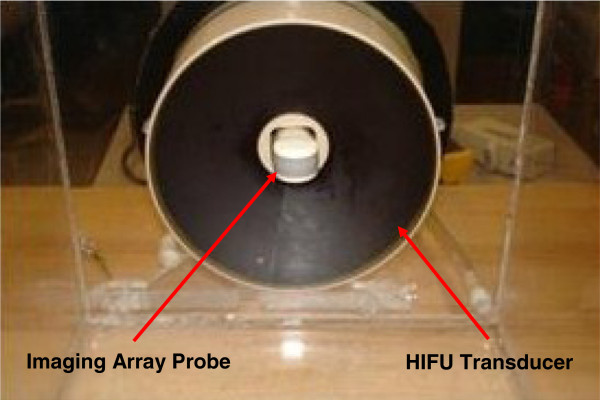
The HIFU transducer and the imaging probe in a confocal arrangement.

Figure 1 illustrates the schematic diagram of the experimental setup. The HIFU transducer was installed inside a water tank filled with degassed and deionized water at room temperature. The tissue sample placed on the tissue holder would then be submerged in the tank at the focal region such that it would cover the entire focal area. The imaging probe was installed confocally through a hole at the center of the HIFU transducer (Figure 2) to ensure that it was always scanning the focal region of the HIFU transducer in the imaging plane.

The thermal effects of the HIFU treatments during the slow HIFU were measured in the tissue samples by monitoring the temperature change during lesion formation using two K-type calibrated thermocouples (made in-house). The calibrated thermocouples were built using 76.2-μm chromel and alumel wires (California Fine Wire, Grover Beach, CA, USA) and then placed inside 22-gauge needles. The thermocouples were implanted at the focal region and the post-focal region of the HIFU beam. The temperature data that were obtained from the focal region and post-focal regions provided information with regard to the extent of thermal damage within the tissue sample. In addition, they were utilized to control the experiment and to ensure that the HIFU beam was only targeting the focal region without causing any thermal damage to the post-focal region.

### Porcine muscles

The experiments were conducted on freshly excised *ex vivo* porcine muscle tissues purchased from a local butcher shop within 6 h of butchering. The muscle specimens used in the slow HIFU experiments were cut and trimmed to a size of around 100 mm × 120 mm × 100 mm and immersed in 0.9% degassed deionized saline solution at 5°C. All experiments were performed within 12 h of purchasing the tissue. Before every experiment, first, all the samples were examined using the conventional B-mode ultrasound images to avoid large layers of fat or cavities in the HIFU acoustic path as this might affect the delivery of HIFU energy to the focal region. In addition, the tissue samples were allowed to reach room temperature while being submerged in the saline solution. Before sonication, the tissue samples were degassed in a desiccator connected to a vacuum pump for 30 min at an absolute pressure of 4 kPa. This procedure has been utilized in other similar studies [34] and has been proven successful in removing air pockets from the tissue. When moving the tissue samples from the desiccator to the tissue holder in the water tank, they were kept submerged in the degassed deionized saline in order to minimize the potential of cavitation generation during HIFU exposure. After HIFU treatment, the samples were sliced along the RF data acquisition plane through the center of the lesion for photo capture and gross pathology examinations.

The muscle specimens used in the 40-s duration HIFU experiments were cut and trimmed to a size of around 20 mm × 80 mm × 100 mm and were prepared for sonication using the same procedure as above except they did not go through the vacuum pumping step. Similarly, after HIFU treatment, the samples were sliced along the RF data acquisition plane through the center of the lesion for photo capture and gross pathology examination.

### HIFU system

The RF signal driving the HIFU transducer was generated by an arbitrary function generator (Model AFG3010; Tektronix, Beaverton, OR, USA). The RF signal was amplified by a class-A broadband RF power amplifier (Model A150; E&I, Rochester, NY, USA), with frequency range of 300 kHz–35 MHz, nominal gain of 53 dB, and class-A nominal linear electric output power of 150 W. The output signal was transferred to the HIFU transducer through a matching network to maximize the transmitted power. A single-element piezo-composite HIFU transducer (Model 6699A101; Imasonic S. A., Voray sur l'Ognon, France) with a resonant frequency of 1 MHz was used throughout this study. The transducer had a 125-mm diameter aperture and a 100-mm geometric focal length. The HIFU transducer output signal was characterized by a calibrated needle hydrophone with an active element diameter of 400 μm (Model HNA-0400; ONDA Corporation, Sunnyvale, CA, USA). In addition to hydrophone measurements, the acoustic field profile of the HIFU transducer was characterized using computer simulations. The computer simulations were conducted using a linear ultrasound simulation software (Linear Acoustic and Temperature Simulation), developed by our research group [35]. The efficiency of the HIFU transducer was defined as the ratio of the converted acoustic power to the transmitted electric power. The transmitted electric power was measured using an electrical power meter (Bird Power Meter 4021; Bird Technologies Group, Cleveland, OH, USA). The acoustic power was measured by a calibrated radiation force balance unit (Model RFB-2000; ONDA Corporation). For the HIFU transducer, the free-field (in water) spatially averaged intensity, *I*_SA_, was estimated using the following equation [36]:

(1) $$I_{\mathrm{SA}}=0.867\frac{P}{D^{2}}$$

where *P* was the TAP measured using the calibrated acoustic power meter at the surface of the transducer, and *D* is the focal beam width at full width at half maximum (FWHM) measured using the calibrated hydrophone.

### Ultrasound imaging system

An ultrasound imaging system (SonixRP® scanner, Ultrasonix Inc., Richmond, Canada) and an endocavity array probe (EC9-5/10, Ultrasonix Inc.) with 128 elements, center frequency of 7 MHz, and bandwidth of 3 MHz were used to acquire backscattered ultrasound RF data. To avoid any interference while acquiring RF backscattered signals during HIFU exposure, the ultrasound imaging system and the function generator were synchronized using a micro-controller (Model M68HC11; Motorola, Inc., Schaumburg, IL, USA).

### RF data acquisition

Backscattered RF data were acquired before, during, and after each HIFU exposure to estimate the initial, transient, and final acoustic properties of tissue. During HIFU treatment, the RF data frames were obtained by briefly turning off the HIFU transducer in order to avoid acoustic and electrical interferences caused by the HIFU transducer.

**Figure 3:**
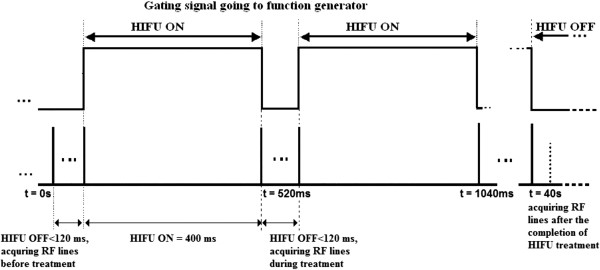
Timing diagram of HIFU exposure and data acquisition.

For the shorter-duration HIFU exposures (40 s), the duration of interruption was 120 ms (off-time) for every HIFU on-time, allowing the capture of two RF data frames, and the HIFU on-time was 400 ms, resulting in a duty cycle of 77%. The total HIFU treatment time was 40 s for TAP levels of 34, 37, 39, 44, and 49 W. A total of four lesions were created at every TAP value. RF data were acquired 10 min after the completion of HIFU treatment, and in one case, RF data were acquired 13 h after the completion of HIFU treatment as well. Figure 3 illustrates the timing diagram of HIFU exposure and RF data acquisition.

During the slow HIFU experiment, the tissue sample was treated with HIFU exposure until the temperature at the focal region, measured by the thermocouple, rose to levels above 60°C. Consequently, the total HIFU treatment time was 9 min and 39 s for TAP of 5 W. RF data were continuously acquired for 9 min after the completion of HIFU treatment (18 min and 39 s in total).

### RF data processing

In this study, a simplifying assumption was made that the frequency-dependent attenuation coefficient of soft tissue, *α*(*f*), increases linearly with frequency [29,34]:

(2) $$\alpha \left(f\right)=\alpha_{0}+\beta \left(f-f_{c}\right)$$

where *α*_0_ was the attenuation coefficient (dB/cm) at the center frequency (*f*_c_) of the transmitted pulse, and *β* was the least squares attenuation slope (dB/MHz.cm). Parameters *α*_0_ and *β* are usually referred to as attenuation intercept and attenuation slope, respectively [26].

In the time domain, for an initial pulse *p*_0_(*t*), the pulse *p*(*t*, *z*) propagating in a medium at time *t* and a distance *z* can be described by using a convolution operator as [37]

(3) $$p\left(t,z\right)=p_{0}\left(t\right)*\text{MIRF}\left(t,z\right)$$

where MIRF is the material impulse response function. Taking the Fourier transfer of both sides results in the following equation in frequency domain:

(4) $$P\left(f,z\right)=P_{0}\left(f\right)\text{MTF}\left(f,z\right)$$

where MTF is the material transfer function in the frequency domain, described by [37],

(5) $$\text{MTF}\left(f,z\right)=\exp\left[\gamma_{T}\left(f\right)z\right]$$

where

(6) $$\gamma_{T}\left(f\right)=-\alpha_{a}\left(f\right)-\mathrm{i\delta}\left(f\right)$$

where *α*_a_ is the frequency-dependent absorption loss and

(7) $$\delta \left(f\right)=k_{0}\left(f\right)+\delta_{E}\left(f\right)$$

where *k*_0_ is the baseline wave number equal to $\frac{\omega }{c_{0}}$, *c*_0_ is the sound speed at the center frequency of the spectrum of the pulse, and *δ*_E_(*f*) is an excess dispersion term required by causality [37].

For the general case of a given pulse *P*_0_(*f*) propagating in a given material over the paths *z*_1_ and *z*_2_,

(8) $$\mathrm{MTF}\left(f,z_{1}+z_{2}\right)=\exp\left[\gamma_{T}\left(f\right)z_{1}\right]\times \exp\left[\gamma_{T}\left(f\right)z_{2}\right],$$

so

(9) $$\mathrm{MTF}\left(f,z_{1}+z_{2}\right)=\text{exp}\left[\gamma_{T}\left(f\right)\left(z_{1}+z_{2}\right)\right].$$

In the specific case of pulse-echo imaging,

(10) $$z_{1}=z_{2}=z.$$

Therefore,

(11) $$\mathrm{MTF}\left(f,2z\right)=\exp\left[2.\gamma_{T}\left(f\right).z\right].$$

The MTF defined in Equation 11 does not take into account the effect of the frequency-dependent scattering (*α*_s_). To compensate for this deficiency, the scattering properties of soft biological tissue were assumed to remain constant before, during, and after the HIFU exposure. The frequency-dependent attenuation coefficient is described as a linear combination of the frequency-dependent scattering coefficient and frequency-dependent absorption coefficient as [38]

(12) $$\alpha \left(f\right)=\alpha_{s}\left(f\right)+\alpha_{a}\left(f\right).$$

Assuming that *α*_s_(*f*) remains constant,

(13) $$\alpha \left(f\right)=C+\alpha_{a}\left(f\right),$$

and by estimating changes in *α*_a_(*f*), changes in *α*(*f*) can be estimated,

(14) $$\alpha_{\text{initial}}\left(f\right)=C+\alpha_{a\_\text{initial}}\left(f\right)$$

(15) $$\alpha_{\text{final}}\left(f\right)=C+\alpha_{a\_\text{final}}\left(f\right)$$

(16) $$\Delta \alpha \left(f\right)=\alpha_{\text{final}}\left(f\right)-\alpha_{\text{initial}}\left(f\right)=\Delta \alpha_{a}\left(f\right).$$

Given the negligible contribution of velocity dispersion within the narrow bandwidths used in this study (approximately 3 MHz), the *δ*(*f*) term of *γ*_T_(*f*) was neglected by discarding the imaginary component of *γ*_T_(*f*).

The attenuation slope algorithm was implemented to estimate Δ*β* by estimating the least squares slope of the line fitted to Δ*α*(*f*). Changes in the least squares slope of the line fitted to Δ*α*(*f*) were constantly estimated throughout the entire treatment cycle with respect to a reference frame obtained right before the HIFU exposure, at *t*_0_ (*t* = 0s). The algorithm would generate a set of two-dimensional matrices, each representing a map of Δ*β* values (as a function of time *t*, depth *z*, and line number), with respect to the reference frame. The following is a mathematical description of the steps implemented in order to estimate Δ*β* for one window of signal with respect to a reference window, at location *z*, in the frequency domain [37].

(17) $$P{\left(f,z\right)}_{\text{signal}}=P_{0}\left(f\right)\text{MTF}\left(f,z\right)$$

(18a) $$\ln P{\left(f,z\right)}_{\text{signal}}=\text{ln}P_{0}\left(f\right)+\text{lnexp}\left[2.\gamma_{T}\left(f\right).z\right]$$

(18b) $$\text{ln}P{\left(f,z\right)}_{\text{signal}}=\ln P_{0}\left(f\right)+2.\gamma_{T}\left(f\right).z$$

(19a) $$S_{\text{signal}}\left(f\right)=-\text{Re}\left\{\text{ln}P_{0}\left(f\right)+2.\gamma_{T}\left(f\right).z\right\}.$$

Substituting *γ*_T_(*f*) = − *α*(*f*) − *iδ*(*f*) in Equation 19a yields

(19b) $$S_{\text{signal}}\left(f\right)=-\text{Re}\left\{\text{ln}P_{0}\left(f\right)\right\}+2.\alpha {\left(f\right)}_{\text{signal}}.z.$$

Similarly, the reference obtained at *t*_0_ (*t* = 0s) is

(20a) $$S_{\text{reference}}\left(f\right)=-\text{Re}\left\{\text{ln}P_{0}\left(f\right)+2.\gamma_{T}\left(f\right).z\right\}$$

(20b) $$\begin{array}{c} S_{\text{reference}}\left(f\right)=-\text{Re}\left\{\text{ln}P_{0}\left(f\right)\right\}\\ \;+2.\alpha_{a}{\left(f\right)}_{\text{reference}}.z. \end{array}$$

Subtracting *S*_reference_ from *S*_signal_ results in

(21a) $$\Delta S\left(f\right)=S_{\text{signal}}\left(f\right)-S_{\text{reference}}\left(f\right)$$

(21b) $$\begin{array}{l} \Delta S\left(f\right)=2.z.\left(\alpha_{a}{\left(f\right)}_{\text{signal}}-\alpha_{a}{\left(f\right)}_{\text{reference}}\right)\\ \;=2.z.\Delta \alpha_{a}\left(f\right). \end{array}$$

Based on Equation 16,

(21c) $$\Delta S\left(f\right)=2.z.\Delta \alpha \left(f\right),$$

substituting *α*(*f*) = *α*_0_ + *β*(*f* − *f*_c_),

(21d) $$\Delta S\left(f\right)=2.z.\left(\Delta \alpha_{0}+\Delta \beta \left(f-f_{c}\right)\right)$$

(21e) $$\Delta S\left(f\right)=2.z.\Delta \alpha_{0}+2.z.\Delta \beta .\left(f-f_{c}\right)$$

Fitting a line to Δ*S*(*f*) and then diving the slope of the fitted line by 2*z* yields Δ*β*.

The attenuation intercept algorithm was implemented to estimate Δ*α*_0_ using identical steps. However, after the acquisition of Δ*S*(*f*) (Equation 21e), the value of Δ*S*(*f*) at the center frequency, *f*_c_, is evaluated as follows:

(22a) $$\mathrm{\Delta S}\left(f_{c}\right)=2.z.\Delta \alpha_{0}+2.z.\Delta \beta .\left(f_{c}-f_{c}\right)$$

(22b) $$\Delta S\left(f_{c}\right)=2.z.\Delta \alpha_{0}$$

(23) $$\frac{\Delta S\left(f_{c}\right)}{2.z}=\frac{2.z.\Delta \alpha_{0}}{2.z}=\Delta \alpha_{0}.$$

Δ*α*_0_ and Δ*β* were estimated using a moving Blackman window function of length 5 *λ*, with $\lambda =\frac{c}{f_{c}}$, where *f*_c_ was the imaging center frequency (during this study *f*_c_ = 4 MHz). The moving window was shifted by (2.5) *λ* at every iteration, resulting in Δ*z* = 0.96 mm.

## Results

### Field profile analysis and efficiency of the HIFU transducer

Based on hydrophone measurements and ultrasound beam computer simulation results, the axial and lateral focal widths of the HIFU beam were measured to be 2 and 8 mm at FWHM, respectively. The computer simulations were conducted for a TAP value of 1W at the surface of the HIFU transducer.

For input electric powers in the range of 0.8 and 157 W, the efficiency of the transducer was measured to be 64%. Table 1 is a summary of the input electric powers with the corresponding TAP values and free-field spatially averaged intensities. *I*_SA_ were estimated using Equation 1 for a duty cycle of 77% and maximum beam width at FWHM of 2 mm.

**Table 1:** **Total acoustic powers and *I***_**SA **_**calculated at corresponding input electric power levels**

**Input electric power (W)**	**Total acoustic power (W)**	***I***_**SA**_**(W/cm**^**2**^**)**
11	5	117
70	34	737
75	37	801
80	39	845
90	44	961
100	49	1,068

### Changes in attenuation slope (Δ*β*)

**Figure 4:**
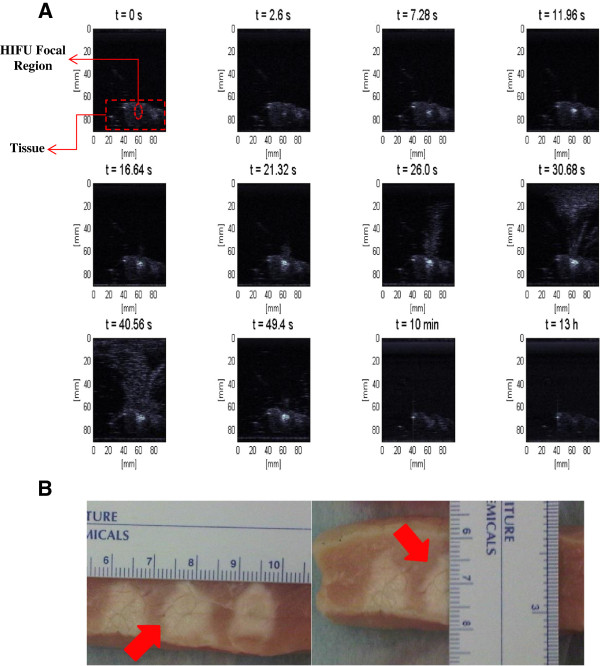
**Lesion growth in *ex vivo *porcine muscle tissue in conventional B-mode images. (A)** The duty cycle was 77%, resulting in TAP of 49 W and average focal intensity of 1,068 W/cm^2^ at the HIFU treatment site, for a total HIFU treatment time of 40 s. **(B)** Axial section of the lesion induced.

**Figure 5:**
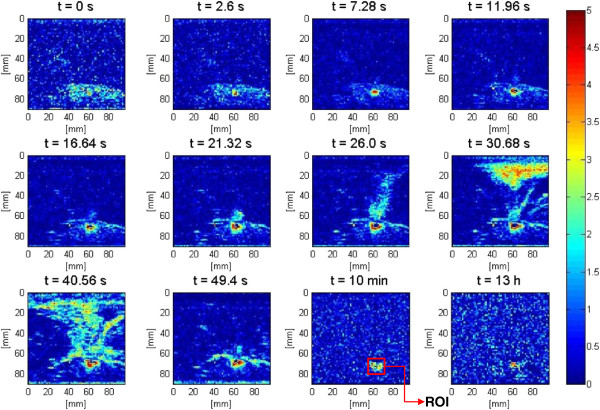
**Lesion growth in *ex vivo *porcine muscle tissue in Δ*β *images.** The duty cycle was 77%, resulting in TAP of 49 W and average focal intensity of 1,068 W/cm^2^ at the HIFU treatment site, for a total HIFU treatment time of 40 s.

The visualization of lesion formation was directly correlated with the B-mode images formed from the pulse-echo RF data, shown in Figure 4A. The induced lesion (Figure 4B) was monitored for 13 h. Figure 5 shows the corresponding Δ*β* images generated using the same RF data, with every frame representing a 2-D map of change in least squares attenuation coefficient slope. As shown in Figure 4A, a bright hyperechoic region appeared at the focal region in the B-mode image at 2.6 s and then enlarged and grew in intensity during HIFU treatment. Corresponding to the hyperechoic region that appeared in the B-mode images, Figure 5 revealed a high-intensity region that appeared in the Δ*β* images at 2.6 s and then enlarged and grew in intensity during HIFU treatment. After the treatment, the bright hyperechoic region in the focal region of the B-mode image began to gradually fade, and after 10 min, it was hardly visible in the B-mode images. After 13 h, the hyperechoic region was virtually invisible in the B-mode image. Meanwhile, even after 13 h, the high-intensity region in the Δ*β* image remained visible. However, this high-intensity region decreased in size and intensity after 10 min had passed, and after 13 h, it decreased in size and intensity to a higher extent.

**Figure 6:**
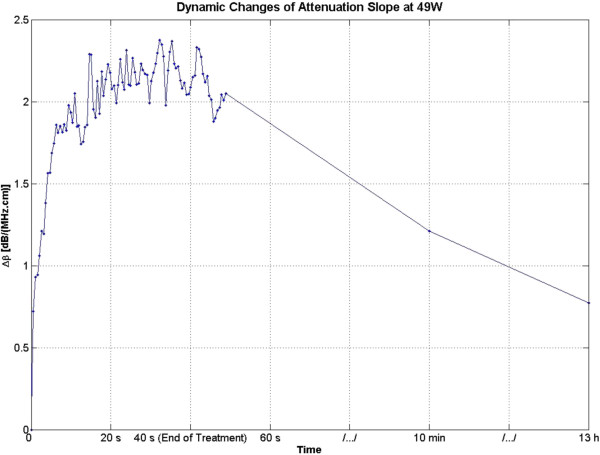
**Dynamic changes of Δ*β *in *ex vivo *porcine muscle tissue during HIFU treatment.** Δ*β* values were estimated by spatially averaging the Δ*β* values axially and laterally in the region of interest (10 mm × 10 mm) centered around the lesion generated by HIFU beam. The duty cycle was 77%, resulting in TAP of 49 W and average focal intensity of 1,068 W/cm^2^ at the HIFU treatment site, for a total HIFU treatment time of 40 s.

Figure 6 shows the dynamic changes of attenuation slope (Δ*β*) in the region of interest. Δ*β* values were estimated by spatially averaging the Δ*β* values axially and laterally in the region of interest (10 mm × 10 mm) centered around the lesion generated by HIFU as shown in Figure 5. As evident in Figure 6, Δ*β* rose very rapidly in the first 20 s of treatment from 0 to 2 dB/(MHz.cm) and stayed at 2 dB/(MHz.cm)with some fluctuations for the next 20 s until the end of HIFU treatment at 40 s. After treatment, Δ*β* gradually decreased, and at 10 min, the Δ*β* algorithm detected a value of 1.3 dB/(MHz.cm). At 13 h, the Δ*β* algorithm detected a value of 0.75 dB/(MHz.cm).

**Figure 7:**
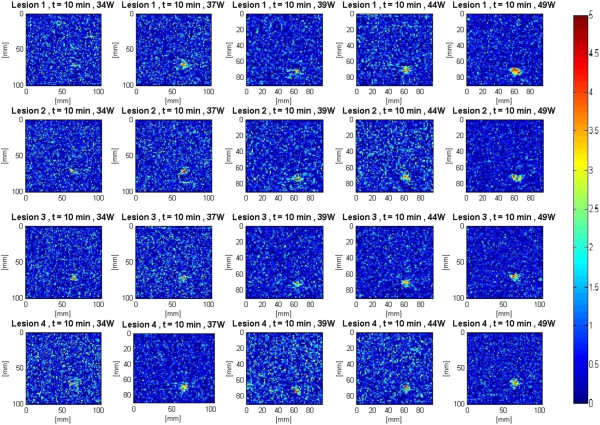
**Δ*β *images detecting lesions in *ex vivo *porcine muscle tissues at 10 min posttreatment.** The duty cycle was 77%, resulting in TAP values of 34, 37, 39, 44, and 49 W, for HIFU treatment time of 40 s. A total of four lesions were created at every power level.

The assumption that all bubble activities had completely vanished 10 min after the end of HIFU treatment [39,40] was made. In addition, due to the heat transfer properties of soft biological tissues, it could be safely assumed that the temperature in the region of interest had cooled down to its pretreatment level. As a result, the output of the Δ*β* algorithm at 10 min was used to assess the performance of the algorithm in detecting tissue thermal damage in the absence of boiling bubbles and temperature rise. Figure 7 shows Δ*β* images generated by the attenuation slope algorithm at different TAP values 10 min after the end of treatment. A total of four lesions were created at every TAP. These Δ*β* images were used to assess the performance of the attenuation slope algorithm.

**Figure 8:**
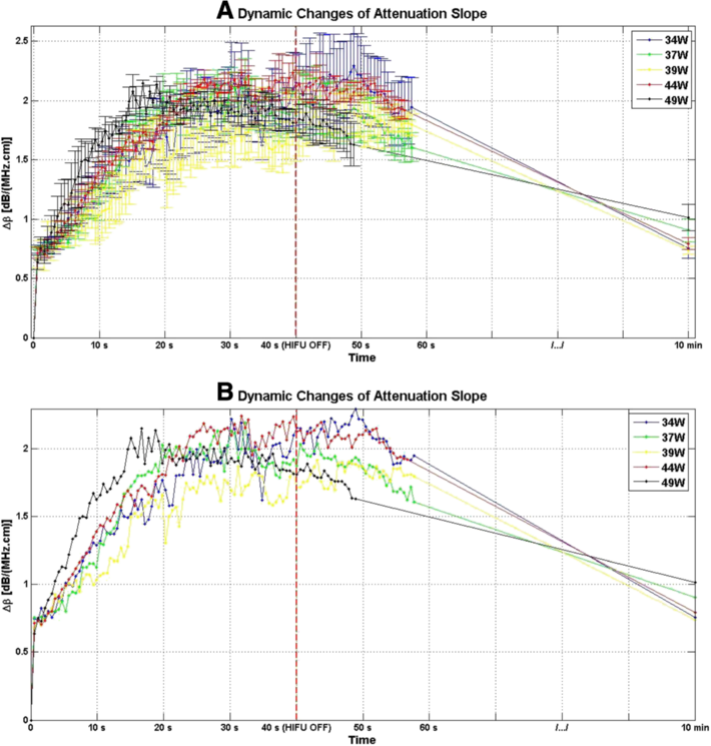
**Dynamic changes of Δ*β *in *ex vivo *porcine muscle tissue during HIFU treatment. (A)** A total of four lesions were created at every TAP. Δ*β* values for each lesion were estimated by spatially averaging the Δ*β* values in ROI, and then those values were further averaged with each other, generating an average profile for each TAP value. For a monitoring duration of 10 min, the duty cycle was 77%, resulting in TAP values of 34, 37, 39, 44, and 49 W, for HIFU treatment time of 40 s. **(B)** Dynamic changes of Δ*β* in *ex vivo* porcine muscle tissue during HIFU treatment presented without the error bars.

Figure 8 shows the dynamic changes of attenuation slope as a result of HIFU treatment in *ex vivo* porcine muscle tissue at different TAP values, for a duration of 10 min. Figure 8 indicates that at all investigated TAP values, Δ*β* rose very rapidly in the first 20 s of treatment, from 0 to values in the range of 1.5–2 dB/(MHz.cm)and then maintained its level within that range with some fluctuations over the next 20 s to the end of HIFU treatment at 40 s. After the end of treatment, Δ*β* values gradually decreased, and after10 min, the Δ*β* algorithm resulted in values in the range of 0.75–1 dB/(MHz/cm).

**Figure 9:**
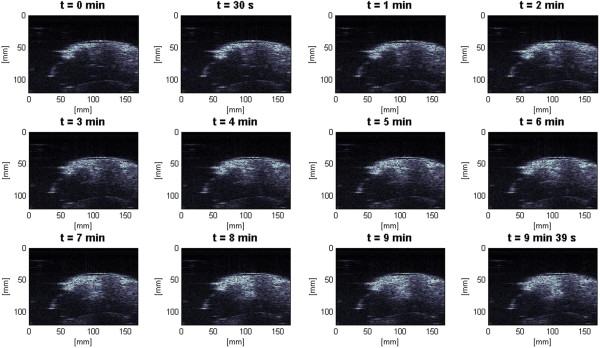
**Lesion growth in *ex vivo *porcine muscle tissue in conventional B-mode images during slow HIFU.** The slow HIFU experiment was conducted with a duty cycle of 77% and TAP of 5 W, resulting in an average focal intensity of 117 W/cm^2^ at the HIFU treatment site, for a total HIFU treatment time of 9 min and 39 s. B-mode images were obtained continuously from the beginning of treatment to the end of treatment.

**Figure 10:**
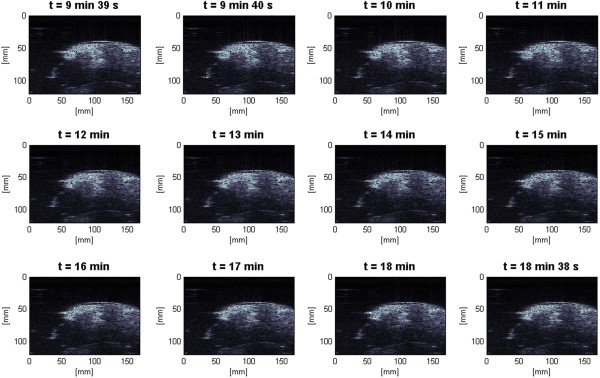
**B-mode images during the cooling sequence.** Induced lesion in *ex vivo* porcine muscle tissue in conventional B-mode images during the cooling cycle of the slow HIFU experiment. The B-mode images were obtained continuously until 9 min after the end of HIFU treatment.

**Figure 11:**
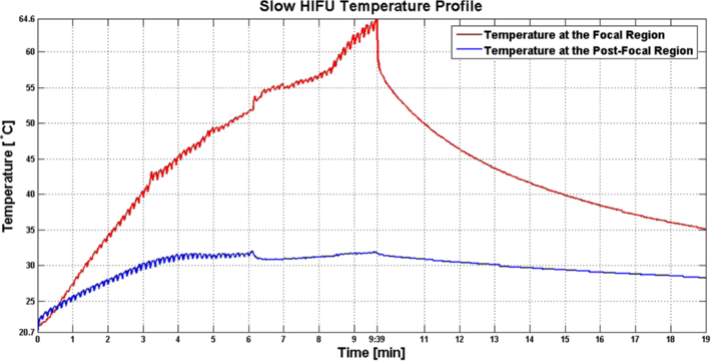
**Temperature profile for the slow HIFU experiment.** Temperature data were collected from the focal and post-focal regions of the tissue sample using two thermocouples for a duration of 19 min.

**Figure 12:**
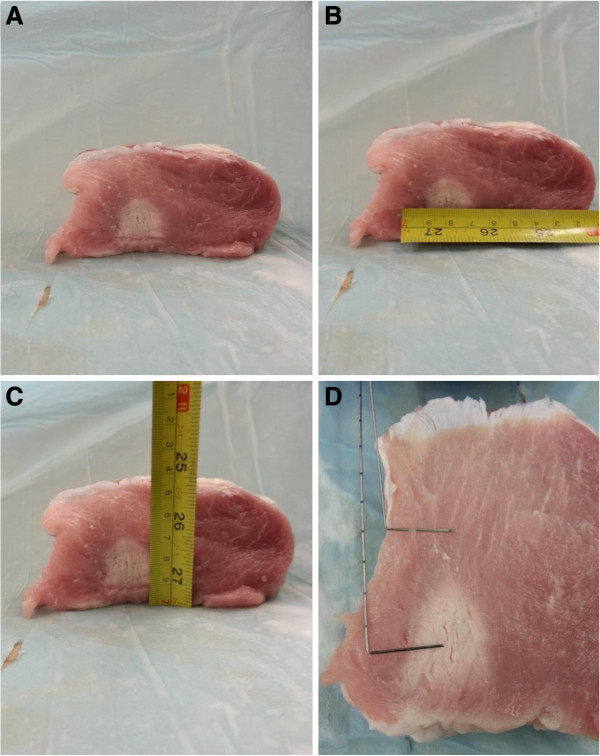
**Induced lesion in *ex vivo *porcine muscle tissue resulting from exposure to slow HIFU. (A, B, C) **Axial section of the lesion induced within the tissue sample, with the HIFU transducer sonicating from the bottom of the tissue. **(D) **A thin slice of tissue sample containing the axial section of the lesion induced, with the thermocouples showing the locations where temperature data were collected.

**Figure 13:**
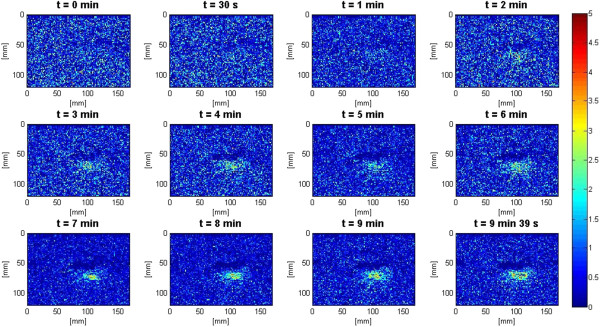
**Lesion growth in *ex vivo *porcine muscle tissue in Δ*β *images during slow HIFU.** The slow HIFU experiment was conducted with a duty cycle of 77% and TAP of 5 W, resulting in an average focal intensity of 117 W/cm^2^ at the HIFU treatment site, for a total HIFU treatment time of 9 min and 39 s.

**Figure 14:**
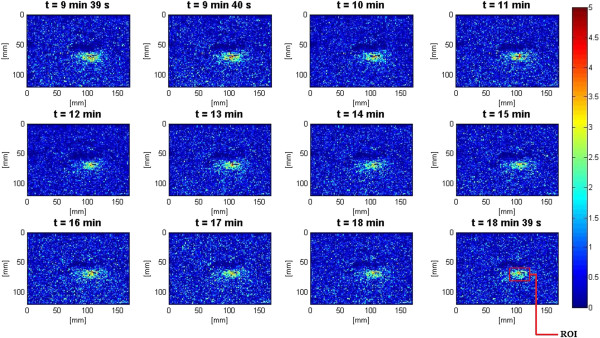
**Δ*β *images during the cooling cycle of the slow HIFU experiment.** Induced lesion in *ex vivo* porcine muscle tissue in Δ*β* images during the cooling cycle of the slow HIFU experiment. The Δβ images were generated until 9 min after the end of the HIFU treatment.

In the slow HIFU experiment, once again the visualization of lesion formation was directly correlated with the B-mode images formed from the pulse-echo RF data. Figure 9 represents the B-mode images obtained during the heating sequence from the start of treatment to the end of treatment at 9 min and 39 s. Figure 10 represents the B-mode images during the cooling sequence obtained until 9 min from the end of the treatment. Figure 11 represents the temperature profile at the focal and post-focal regions of the tissue sample, and Figure 12 represents the induced lesion. Figures 13 and 14 show the corresponding Δ*β* images generated using the same RF data. Unlike in Figure 4A, the bright hyperechoic region at the focal region in the B-mode image was not present. However, once again, Figures 13 and 14 revealed a high-intensity region that appeared in the Δ*β* images at 3 min and then enlarged and grew in intensity during HIFU treatment. The high-intensity region in the Δ*β* images remained visible during the cooling cycle. However, it slightly decreased in size and intensity after 9 min had passed.

**Figure 15:**
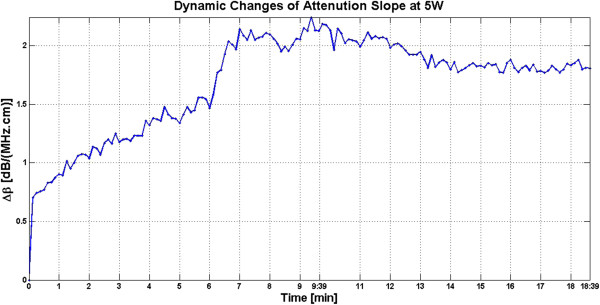
**Dynamic changes of Δ*β *in *ex vivo *porcine muscle tissue during slow HIFU treatment.** Δ*β* values were estimated by spatially averaging the Δ*β* values axially and laterally in the region of interest (20 mm × 34 mm) centered around the lesion generated by HIFU as shown in Figure 14. The duty cycle was 77%, with TAP of 5 W, resulting in an average focal intensity of 117 W/cm^2^ at the HIFU treatment site, for a total HIFU treatment time of 9 min and 39 s.

Figure 15 shows the dynamic changes of attenuation slope (Δ*β*) in the region of interest during the slow HIFU exposure. Δ*β* maps were generated by spatially averaging the Δ*β* values axially and laterally in the region of interest (20 mm × 34 mm) centered around the lesion generated by HIFU as shown in Figure 14. As evident in Figure 15, Δ*β* rose gradually in the first 6 min of treatment from 0 to 1.5 dB/(MHz.cm). From 6 to 7 min, there was a jump in the value of Δ*β* from 1.5 to 2.1 dB/(MHz.cm). Δ*β* maintained its value around 2.1 dB/(MHz.cm) with some fluctuations until the end of HIFU treatment at 9 min and 39 s when it reached a maximum value of 2.2 dB/(MHz.cm). After treatment, Δ*β* gradually decreased to 1.8 dB/(MHz.cm)and remained stable at that value. Once again, the output of the Δ*β* algorithm at the end of the treatment was used to assess the performance of the algorithm in detecting tissue thermal damage in the absence of boiling bubbles and temperature rise. Table 2 summarizes the resulting Δ*β* values in the region of interest (ROI), for different TAP values, approximately 10 min after HIFU treatment.

**Table 2:** **Δ** ***β *** **values vs. total acoustic power at *t *** **= 10 min**

**Total acoustic power (W)**	**Δ** ***β*** **(dB/(MHz.cm))**
5	1.81 ± 0.05
34	0.76 ± 0.09
37	0.91 ± 0.10
39	0.74 ± 0.03
44	0.79 ± 0.05
49	1.01 ± 0.11

### Changes in attenuation intercept (Δ*α*_0_)

**Figure 16:**
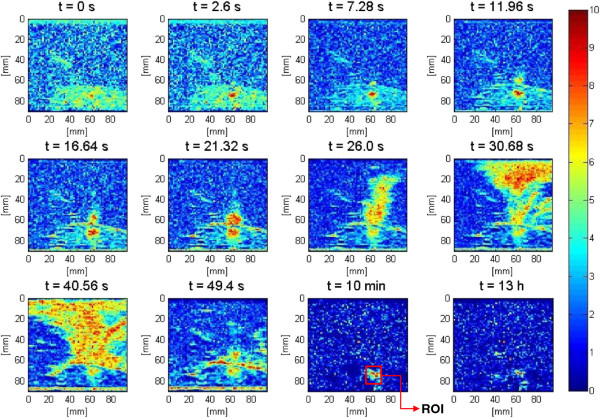
**Lesion growth in *ex vivo *porcine muscle tissue in Δ*α***_**0 **_**images.** The duty cycle was 77%, resulting in TAP of 49 W and average focal intensity of 1,068 W/cm^2^ at the HIFU treatment site, for a total HIFU treatment time of 40 s.

**Figure 17:**
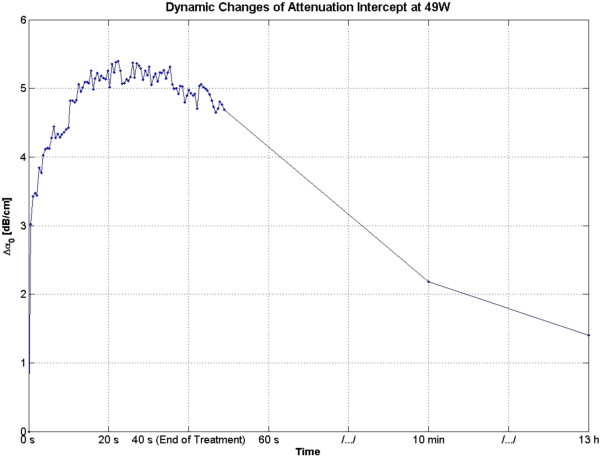
**Dynamic changes of Δ*α***_**0 **_**in *ex vivo *porcine muscle tissue during HIFU treatment.** Δ*α*_0_ values were estimated by spatially averaging the Δ*α*_0_ values axially and laterally in the region of interest (10 mm × 10 mm) centered around the lesion generated by HIFU as shown in Figure 8. The duty cycle was 77%, resulting in TAP of 49 W and average focal intensity of 1,068 W/cm^2^ at the HIFU treatment site, for a total HIFU treatment time of 40 s.

The attenuation intercept algorithm was used on the same set of RF data that was used for the attenuation slope algorithm in the previous section. Once again the visualization of lesion formation was directly correlated with the B-mode images (Figure 4). Figure 16 shows the corresponding Δ*α*_0_ images, with every frame representing a 2-D map of change in attenuation coefficient intercept (Δ*α*_0_). Similar to previous results, corresponding to the hyperechoic region that appeared in the B-mode images at *t* = 2.6 s, a high-intensity region appeared in the Δ*α*_0_ images at 2.6 s (Figure 16). Once again this high-intensity region enlarged and grew in intensity during HIFU treatment. Thirteen hours after the treatment, the high-intensity region in the Δ*α*_0_ images still remained visible. In an outcome similar to Δ*β* images, the high-intensity region decreased in size and intensity after 10 min had passed, and after 13 h, it decreased in size and intensity to a higher extent. Δ*α*_0_ values were estimated by spatially averaging the Δ*α*_0_ values axially and laterally in the region of interest (10 mm × 10 mm) centered around the lesion generated by HIFU as shown in Figure 16. Based on Figure 17, Δ*α*_0_ rose very rapidly in the first 20 s of treatment, from 0 to 5 dB/cm and then stayed at 5 dB/cm with some fluctuations over the next 20 s to the end of HIFU treatment at 40 s. After treatment, Δ*α*_0_ gradually decreased, and at 10 min posttreatment, the Δ*α*_0_ algorithm resulted in a value of approximately 2 dB/cm. At 13 h, the Δ*α*_0_ algorithm resulted in a value of 1.5 dB/cm.

**Figure 18:**
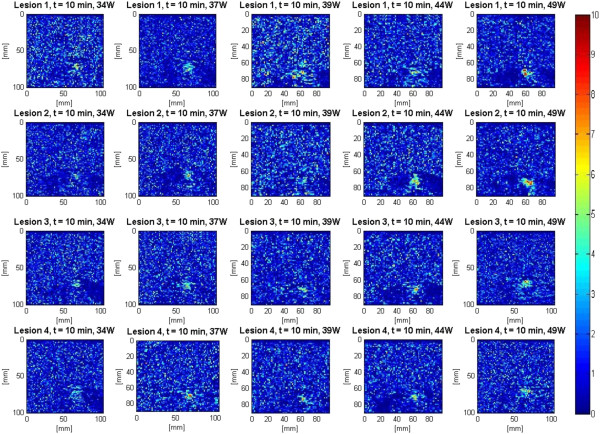
**Δ*α***_**0 **_**images detecting lesions in *ex vivo *porcine muscle tissues at 10 min post treatment.** The duty cycle was 77%, resulting in TAPs of 34, 37, 39, 44, and 49 W for a HIFU treatment time of 40 s. A total of four lesions were created at every power level.

Figure 18 shows Δ*α*_0_ images generated by the attenuation intercept algorithm at different TAP values 10 min after the end of treatment. These Δ*α*_0_ images were used to assess the performance of the attenuation intercept algorithm.

**Figure 19:**
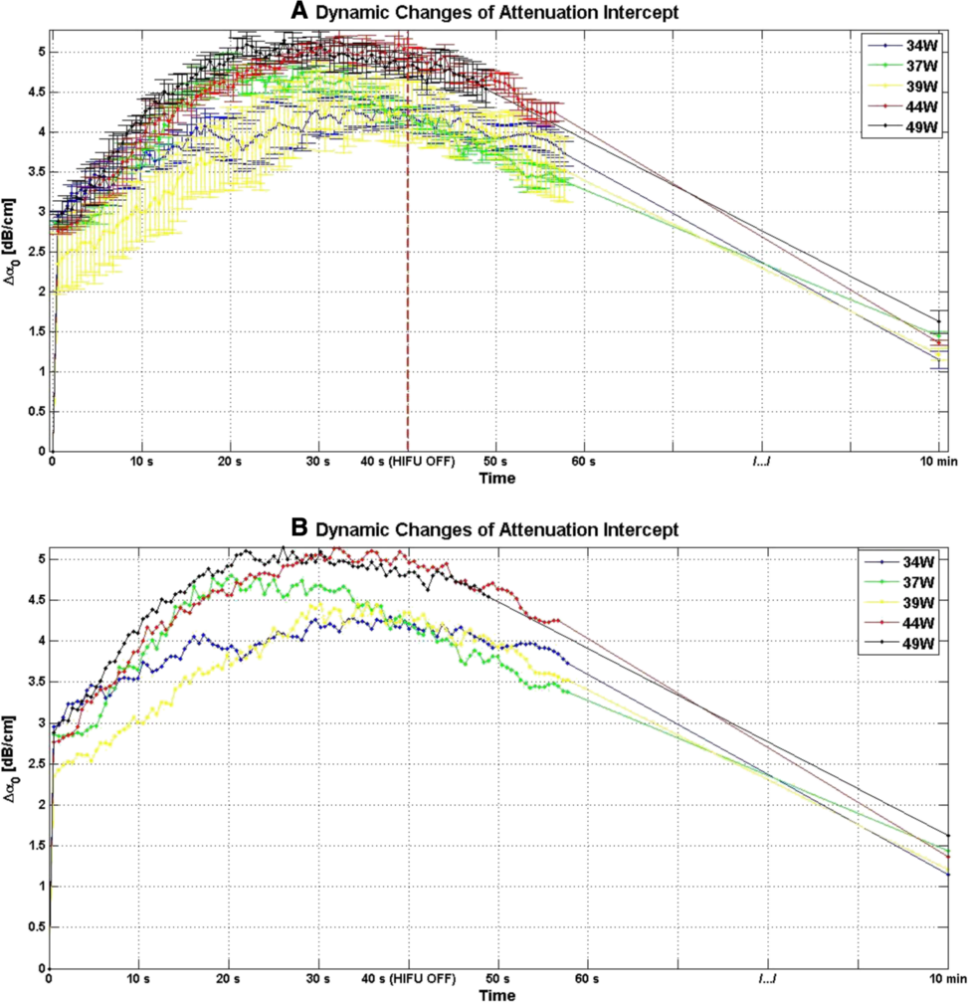
**Dynamic changes of Δ*α***_**0 **_**in *ex vivo *porcine muscle tissue during HIFU treatment. (A)** A total of four lesions were created at every TAP. Δ*α*_0_ values for each lesion were estimated by spatially averaging the Δ*α*_0_ values in the ROI, and then those values were further averaged with each other generating an average profile for each TAP value. For monitoring duration of 10 min, the duty cycle was 77%, resulting in TAP values of 34, 37, 39, 44, and 49 W, for a HIFU treatment time of 40 s. **(B)** Dynamic changes of Δ*α*_0_ in *ex vivo* porcine muscle tissue during HIFU treatment presented without the error bars.

Figure 19 shows the dynamic changes of attenuation intercept as a result of HIFU treatment in *ex vivo* porcine muscle tissue at different TAP values. As evident in Figure 19, at all the investigated TAP values, Δ*α*_0_ rose very rapidly in the first 20 s of treatment, from 0 to somewhere in the range of 4–5 dB/cm and then maintained its value within that range with some fluctuations over the next 20 s to the end of HIFU treatment at 40 s. After the end of treatment, Δ*α*_0_ gradually decreased, and after10 min, the Δ*α*_0_ algorithm resulted in values in the range of 1–1.6 dB/cm.

**Figure 20:**
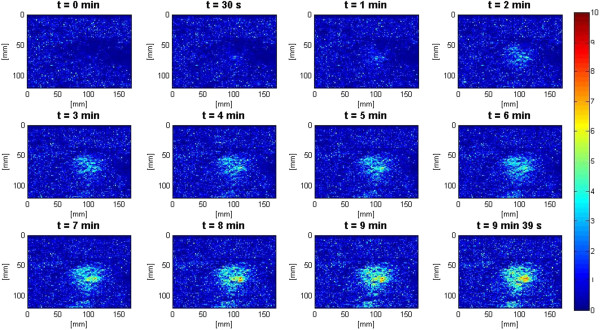
**Lesion growth in *ex vivo *porcine muscle tissue in Δ*α***_**0 **_**images during slow HIFU experiment.** The slow HIFU experiment was conducted with a duty cycle of 77% and TAP of 5 W, resulting in an average focal intensity of 117 W/cm^2^ at the HIFU treatment site, for a total HIFU treatment time of 9 min and 39 s.

**Figure 21:**
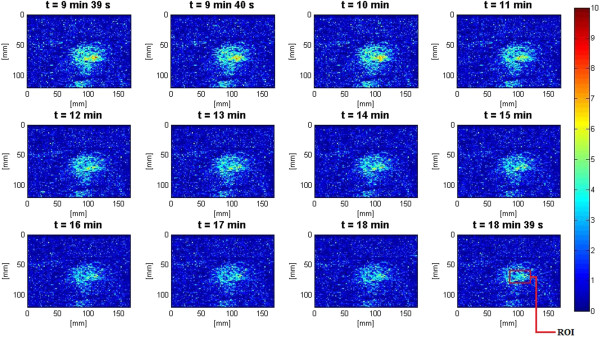
**Δ*α***_**0 **_**images during the cooling cycle of the slow HIFU experiment.** Induced lesion in *ex vivo* porcine muscle tissue in Δ*α*_0_ images during the cooling cycle of the slow HIFU experiment. The Δ*β* images were generated until 9 min after the end of the HIFU treatment.

**Figure 22:**
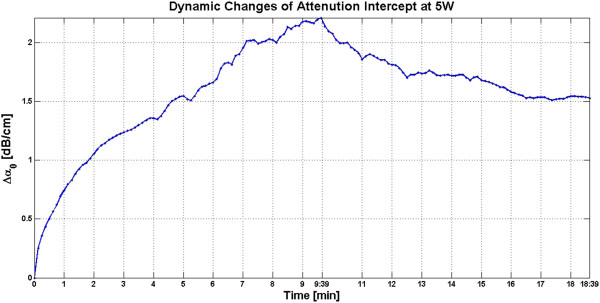
**Dynamic changes of Δ*α***_**0 **_**in *ex vivo *porcine muscle tissue during slow HIFU treatment.** Δ*α*_0_ values were estimated by spatially averaging the Δ*α*_0_ values axially and laterally in the region of interest (20 mm × 34 mm) centered around the lesion generated by HIFU as shown in Figure 14. The duty cycle was 77% with TAP of 5 W, resulting in an average focal intensity of 117 W/cm^2^ at the HIFU treatment site, for a total HIFU treatment time of 9 min and 39 s.

In the slow HIFU experiment, once again the visualization of lesion formation was directly correlated with the B-mode images formed from the pulse-echo RF data. Figures 20 and 21 show the corresponding Δ*α*_0_ images generated using the same RF data during the slow HIFU treatment experiment. Once again, Figures 20 and 21 revealed a high-intensity region that appeared in the Δ*α*_0_ images at 3 min and then enlarged and grew in intensity during HIFU treatment. The high-intensity region in the Δ*α*_0_ images remained visible during the cooling cycle and revealed minor amounts of decrease in size and intensity after 9 min had passed. Figure 22 shows the dynamic changes of attenuation intercept (Δ*α*_0_) in the region of interest. Δ*α*_0_ maps were generated by spatially averaging the Δ*α*_0_ values axially and laterally in the region of interest (20 mm × 34 mm) centered around the lesion generated by HIFU as shown in Figure 21. As evident in Figure 22, Δ*α*_0_ rose gradually in the first 6 min of treatment from 0 to 1.7 dB/cm. From 6 to 7 min, there was a jump in the value of Δ*β* from 1.7 to 2 dB/cm. Δ*α*_0_ maintained its value around 2 dB/cm with minor fluctuations until the end of HIFU treatment at 9 min and 39 s when it reached a maximum value of 2.2 dB/cm. After treatment, Δ*α*_0_ gradually decreased to 1.5 dB/cm and remained stable at that value. Once again, the output of the Δ*α*_0_ algorithm at the end of the treatment was used to assess the performance of the algorithm in detecting tissue thermal damage in the absence of boiling bubbles and temperature rise. Table 3 summarizes the resulting Δ*α*_0_ values in the ROI, for different TAP values, approximately 10 min after HIFU treatment.

**Table 3:** **Δ** ***α***_**0 **_**values vs. total acoustic power at *t *** **= 10 min**

**Total acoustic power (W)**	**Δ** ***α***_**0**_**(dB/cm)**
5	1.53 ± 0.07
34	1.15 ± 0.11
37	1.44 ± 0.05
39	1.21 ± 0.07
44	1.36 ± 0.03
49	1.62 ± 0.14

### Attenuation maps vs. conventional B-mode images

**Figure 23:**
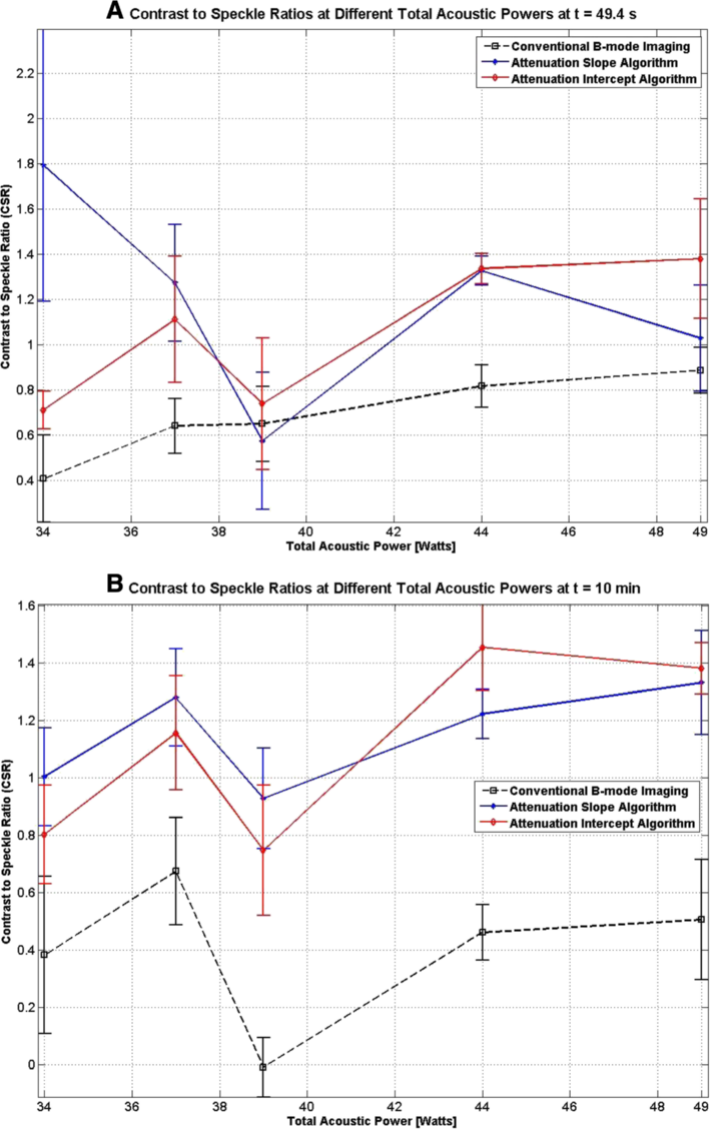
**Comparison of contrast-to-speckle ratios.** CSR values were calculated at various TAP values at **(A)** 10 s and at **(B)** 10 min after the end of HIFU treatment.

To quantitatively compare the performance of the attenuation slope (Δ*β*) and attenuation intercept (Δ*α*_0_) algorithms with each other and with conventional B-mode imaging, the contrast-to-speckle ratios (CSR) [41] of these three different modes of generating images in this study were investigated. The CSR of an image is defined as [41]

(24) $$\text{CSR}=\frac{S_{\text{in}}-S_{\text{out}}}{\sqrt{\sigma_{\text{in}}^{2}+\sigma_{\text{out}}^{2}}}$$

where *S*_in_ is the mean signal measured inside the region of interest, *S*_out_ is the mean signal measured from same-sized regions outside the region of interest, and *σ*^2^_in_ and *σ*^2^_out_ represent the variances of the signal within and outside the region of interest, respectively [41]. CSR values were calculated for the frames that were acquired 10 s after the end of HIFU treatment and for frames that were acquired 10 min after the end of treatment (Figure 23).

For the slow HIFU experiment, the CSR values were similarly calculated at 10 s after the end of the HIFU treatment and 9 min after the end of treatment. The results were separately tabulated in Table 4.

**Table 4:** Contrast-to-speckle ratios at TAP of 5 W and exposure duration of 9 min and 39 s

**Mode of generating image**	**CSR at 10 s after the end of treatment**	**CSR at 10 min after the end of treatment**
Attenuation slope algorithm (Δ*β* image)	0.65	0.49
Attenuation intercept algorithm (Δ*α*_0_)	0.81	0.48
Conventional B-mode imaging	0.47	0.21

## Discussions

It has been postulated that bubbles are usually formed soon after the beginning of HIFU exposure at the HIFU treatment site [3,42]. These bubbles are either induced due to acoustic cavitation effects when the temperatures are still not high enough (referred to as cavitation bubbles) or due to boiling effects when the temperatures are sufficiently high (referred to as boiling bubbles) [3,42]. Boiling bubbles are highly scattering and sufficiently large that in ultrasound B-mode images they are clearly visible as bright hyperechoic regions at the HIFU treatment site [39,42]. Previous studies on transparent tissue-mimicking phantoms have shown the presence of violent bubble activities at the HIFU treatment site mainly due to boiling effects [34]. In our study, at higher HIFU intensities (737–1,068 W/cm^2^), as soon as the HIFU was turned on, bright hyperechoic regions appeared in the B-mode images and then faded rapidly after HIFU was turned off (Figure 4A). Given the intensities at the HIFU treatment site and the fact that the tissue samples used in those experiments were not vacuum pumped, the bubble activities were probably mainly due to boiling effects.

During HIFU treatment, significant increases in values of *β* and *α*_0_ were observed in lesions created in *ex vivo* porcine muscle tissues. These results agreed with previous results by similar studies [34]. The presence of bubble activities was detected simultaneously with the presence of hyperechoic regions in the B-mode images. Therefore, the rapid increases in Δ*β* and Δ*α*_0_ and their consequent fluctuations (Figures 8 and 19) may be attributed to bubble activities causing abrupt changes in acoustic properties of the tissue at the HIFU treatment site. In one possible scenario, the formation of bubbles at the HIFU treatment site may have amplified the tissue ultrasound scattering properties (as evidenced by the presence of hyperechoic regions). This rapid increase in scattering of tissue at the HIFU treatment site significantly undermines the simplifying assumption in our method that scattering properties would remain constant, leading to a rapid increase in the total attenuation of tissue at that location (Equation 12). Finally, it has been shown that as a result of temperature rise, tissue attenuation coefficient increases [24]. Therefore, the rapid temperature rise at the site of HIFU treatment may have contributed to the rapid increases in Δ*β* and Δ*α*_0_ values as well.

After HIFU treatment, the hyperechoic regions in the B-mode images rapidly faded. Corresponding to B-mode images, the high-intensity regions in Δ*β* and Δ*α*_0_ images began to decrease in size at a relatively slower rate, until they became stable at around 10 min after the end of treatment. This decrease in size may be attributed to the absence of bubbles long after the HIFU exposure and to the fact that the temperature at the site of HIFU exposure cools down to pretreatment levels. Thus, the high-intensity regions detected in Δ*β* and Δ*α*_0_ images at 10 min could be used as true representations of the boundaries of the coagulated regions of the tissue.

In the case of the lesion that was monitored overnight, it was observed that in Δ*β* and Δ*α*_0_ images, the high-intensity region further decreased in size and intensity from 10 min to 13 h after the end of HIFU treatment. This might be attributed to the fact that from 10 min to 13 h, the temperature of the entire tissue and the focal spot significantly cooled down to below pretreatment levels. In addition, the tissue might have absorbed water from the water bath, resulting in changes in scattering properties of the tissue and the site of thermal damage and undermining our initial simplifying assumption regarding *α*_s_.

During the slow HIFU experiment, the hyperechoic regions in the B-mode images did not appear at the same intensity and extent as before. This could be attributed to the special degassing process that was used and the significantly lower HIFU intensity at the treatment site (117 W/cm^2^).

During slow HIFU treatment, once again significant increases in values of *β* and *α*_0_ were observed in the lesion created in *ex vivo* porcine muscle tissues. However, due to the significantly lower HIFU intensity and the special degassing process, the increases in Δ*β* and Δ*α*_0_ and their consequent fluctuations (Figures 15 and 22) can no longer be attributed to bubble activities.

Previous studies have shown that temperature rise affects tissue attenuation coefficient and tissue absorption at temperatures above 50°C [24]. Based on the temperature data (Figure 11) at 6 min after the beginning of HIFU treatment, temperature at the HIFU treatment site was 50°C. However, despite the fact that temperature levels were below 50°C, as Figures 13, 15, 20, and 22 revealed, there were significant increases in values of *β* and *α*_0_ at the ROI from 0 to 6 min after treatment. Therefore, the temperature rise at the site of HIFU treatment could not have contributed to the increases in Δ*β* and Δ*α*_0_ values.

After HIFU treatment, the high-intensity regions in Δ*β* and Δ*α*_0_ images began to decrease in size and intensity at a relatively slow rate, until they became stable at around 9 min after the end of treatment. This decrease in size and intensity may be attributed to the decrease in temperature at the site of the HIFU exposure to levels below 50°C at 2 min after the end of treatment (Figure 11).

In the absence of bubble activities and variations in tissue attenuation coefficient as a function of temperature, it could be concluded that what was being measured by the attenuation slope and attenuation intercept algorithms were changes in acoustic properties of the tissue that were induced due to tissue thermal damage alone. Once again, the high-intensity regions detected in Δ*β* and Δ*α*_0_ images at 9 min after the end of treatment were used as true representations of the boundaries of the coagulated regions of the tissue.

Based on the contrast-to-speckle ratios (Figure 23 and Table 4), there is not enough evidence to suggest that there is a significant difference between the performances of the two algorithms. In comparison to conventional B-mode imaging, in the absence of bubbles and temperature elevations (10 min after the HIFU treatment), the attenuation algorithms significantly outperformed the conventional B-mode images. It was initially expected that, in the presence of bubble activities and temperature elevations, conventional B-mode images would outperform the attenuation algorithms. However, CSR values obtained within 10 s (*t* = 49.4 s) after HIFU treatment showed no significant differences between conventional B-mode imaging and Δ*β* and Δ*α*_0_ maps even in the presence of bubble activities and temperature elevations.

**Figure 24:**
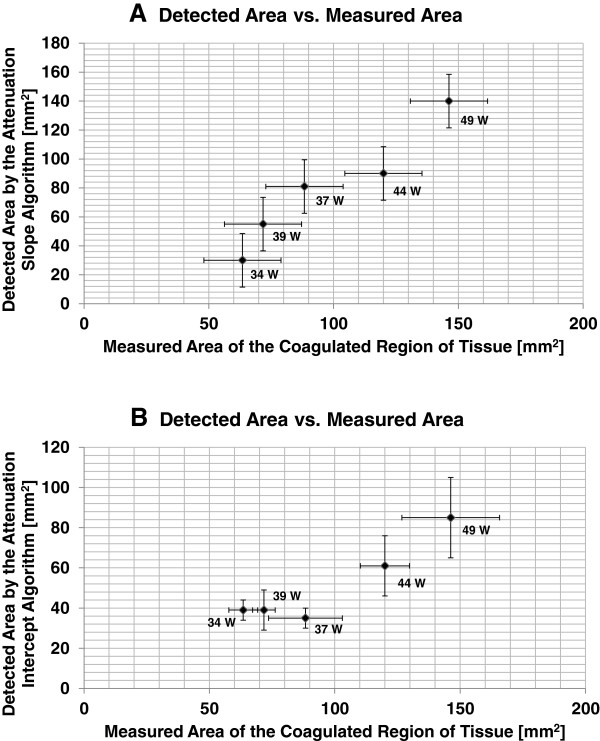
**Comparison of the measured area of the coagulated region vs. the detected area. (A)** Measured area of the coagulated region of the tissue vs. the detected area by the attenuation slope algorithm at *t* = 10 min. **(B)** Measured area of the coagulated region of the tissue vs. the detected area by the attenuation intercept algorithm at *t* = 10 min.

The spatial extent of the detected changes in tissue attenuation coefficient parameters were quantitatively compared with the spatial extent of the resulting thermal lesions by plotting the sizes of the detected lesions (at 10 min after the end of treatment) against the measured sizes of the resulting regions of thermal damage (Figure 24). Based on the results shown in Figure 24, the dimension of the lesions detected by both algorithms correlated with the measured sizes of the actual thermal lesions induced within the tissue samples.

In this study, to minimize motion variances and artifacts, the tissue samples were mounted on an acrylic tissue holder with an acoustic window to minimize tissue movements. Furthermore, while investigating the dynamic changes of Δ*β* and Δ*α*_0_, the values were spatially averaged in the region of interest to minimize the effects of any variances. However, slight tissue movements due to the acoustic radiation force, especially at higher powers during HIFU exposure, were always present to some extent, resulting in movements of the RF data acquisition plane and inducing variances and artifacts in differential Δ*β* and Δ*α*_0_ images. As Figure 4A revealed, starting at 11 s after the beginning of treatment, the HIFU radiation force began to push the tissue at the water tissue boundary along *z* = 60 mm. This effect became more obvious in the rest of the snapshots at 21.3 s, 49.4 s, 10 min, and 13 h. This tissue movement induced artifacts in the Δ*β* images. These artifacts can be seen in the form of voids in the Δ*β* images at the corresponding times (Figure 5).

## Conclusions

We have obtained preliminary data for the changes in attenuation coefficients induced in *ex vivo* porcine muscle tissues due to HIFU coagulation. Changes in least squares attenuation coefficient slope (Δ*β*) and attenuation coefficient intercept (Δ*α*_0_) were both shown to potentially be reliable indicators of tissue thermal damage caused by HIFU exposure. Based on a simplified model, Δ*β* and Δ*α*_0_ values for any given location were estimated as functions of time and location, with respect to pretreatment values of *β* and *α*_0_ at the same location, before, during, and after HIFU treatment at different total acoustic powers, using pulse-echo ultrasound RF data. The rapid increases in attenuation slope and intercept were generally accompanied by some fluctuations due to rapid rises in tissue temperature and the bubble activities in the HIFU focal region. Violent bubble activities were evident as hyperechoic regions in the B-mode images at the HIFU treatment sites. The performance of B-mode images relied more on the effects of bubble activities compared to the Δ*β* and Δ*α*_0_ images. The dynamic changes of attenuation coefficient parameters (Δ*β* and Δ*α*_0_) may be employed in the development of real-time monitoring and guidance of HIFU therapies and evaluation of HIFU-induced lesions.

At this point, further studies are necessary to determine the relative contributions of bubble activities and thermal tissue damage to the dynamic changes in attenuation coefficient parameters (Δ*β* and Δ*α*_0_) in HIFU-induced lesions. To this end, a cavitation detection apparatus could be added to the current experimental setup. Such a system will provide accurate quantitative information on bubble activities (changes in subharmonic and broadband noise), leading to more accurate investigations of dynamic changes of attenuation coefficients during HIFU treatment. Finally, instead of relying on the set of simplifying assumptions, further investigations need to be carried out to study the possibility of expanding the material transfer function so that it will include the effects of changes in scattering properties of tissue.

## Abbreviations

CSR: Contrast-to-speckle ratio; FWHM: Full width at half maximum; HIFU: High-intensity focused ultrasound; MIRF: Material impulse response function; MRI: Magnetic resonance imaging; MTF: Material transfer function; RF: Radio frequency; ROI: Region of interest; TAP: Total acoustic power.

## Competing interests

Both authors declare that they have no competing interests.

## Authors' contributions

SR and JT had equal contributions in conceiving of and designing the research work, carrying out the experiments, analyzing the results, and writing the manuscript. Both authors read and approved the final manuscript.
